# Optimized single-cell RNA sequencing protocol to study early genome activation in mammalian preimplantation development

**DOI:** 10.1016/j.xpro.2023.102357

**Published:** 2023-06-13

**Authors:** Nina Boskovic, Gamze Yazgeldi, Sini Ezer, Mari H. Tervaniemi, Jose Inzunza, Spyridon Panagiotis Deligiannis, Barış Yaşar, Tiina Skoog, Kaarel Krjutškov, Shintaro Katayama, Juha Kere

**Affiliations:** 1 Department of Biosciences and Nutrition, Karolinska Institutet, 14183 Huddinge, Sweden; 2 Department of Obstetrics and Gynecology, University of Helsinki, 00290 Helsinki, Finland; 3 Folkhälsan Research Center, 00290 Helsinki, Finland; 4 Stem Cells and Metabolism Research Program, University of Helsinki, 00290 Helsinki, Finland; 5 Competence Centre of Health Technologies, 50411 Tartu, Estonia; 6 Department of Obstetrics and Gynecology, Institute of Clinical Medicine, University of Tartu, 50406 Tartu, Estonia; 7 Department of Biotechnology, Institute of Molecular and Cell Biology, University of Tartu, 51010 Tartu, Estonia

**Keywords:** Bioinformatics, Sequence analysis, Single Cell, Developmental biology, Genomics, Sequencing, RNA-seq, Molecular Biology, Gene Expression

## Abstract

Here, we present a modification of single-cell tagged reverse transcription protocol to study gene expression on a single-cell level or with limited RNA input. We describe different enzymes for reverse transcription and cDNA amplification, modified lysis buffer, and additional clean-up steps before cDNA amplification. We also detail an optimized single-cell RNA sequencing method for handpicked single cells, or tens to hundreds of cells, as input material to study mammalian preimplantation development.

For complete details on the use and execution of this protocol, please refer to Ezer et al.^1^

## Highlights

• Multiplexed RNA-seq library protocol for single embryos or cells
• 5′ end targeted sequencing for transcription start site capture
• Read normalization by spike-in RNAs for accurate quantification of expression
• Degrading RNAs distinguished by 3′ predominance of read start sites

## Abstract (editor highlights)

Publisher’s note: Undertaking any experimental protocol requires adherence to local institutional guidelines for laboratory safety and ethics.

## Graphical abstract

**Figure:**
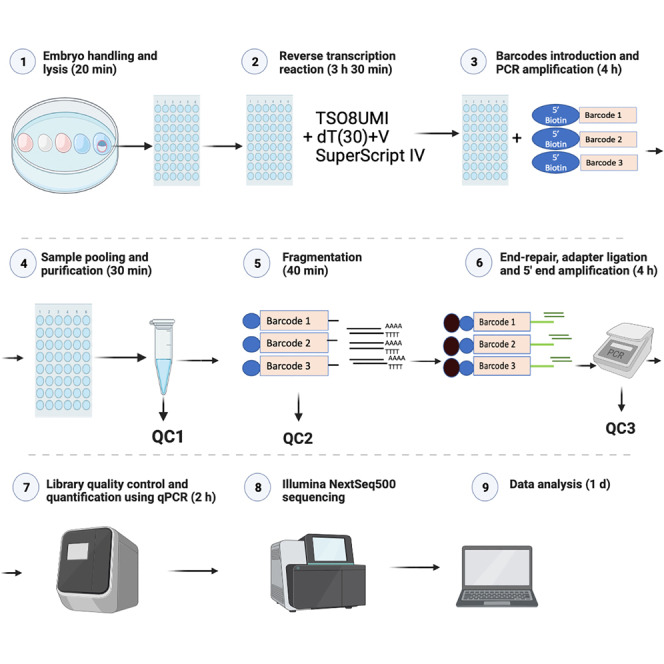

## Before you begin

Single-cell Tagged Reverse Transcription (STRT-Seq) was one of the first multiplexed single cell RNA sequencing (scRNAseq) methods focusing on the 5′ end of mRNA, enabling the study of transcription start sites (TSS).^2^^,^^3^ Since its introduction, the method has been modified to work on the new sequencing platforms such as Illumina NextSeq system, as well as red blood cell-containing, globin-rich tissue samples.^1^ For these protocols, 20–40 ng of template RNA is required, making them suboptimal for the limited amount of mRNA that is present in single cells. Even though high-throughput scRNAseq platforms are now available,^4^ they are limited to situations where at least hundreds or thousands of cells are available, making them unsuitable for analyzing, e.g., mammalian oocytes and embryos.

It is estimated that a human oocyte contains 55 pg of mRNA.^5^ Therefore, we modernized the previous STRT protocols to improve the sensitivity of the method and named the new protocol STRT-N. Our modifications include different reverse transcriptase and cDNA amplification enzymes, modified cell lysis buffer, and additional clean-up step prior to the cDNA amplification.

A STRT-N library consists of 48 barcoded cDNA samples that can be a single cell or a single embryo that consists of 100 cells at blastocyst stage, avoiding batch effects between the samples. In addition to highly consistent quantification of expression, the reads are obtained from the 5′ end of mRNA, thus allowing the detection of transcription start sites (TSS) and alternative gene promotors. In addition, if reads obtained from the preimplantation embryos are at the 3′-UTR of the known protein coding genes, then those are likely degraded maternal transcripts and not translated into proteins. However, most RNA sequencing methods cannot distinguish whether reads at the 3′-UTR are derived from intact transcripts or from degraded ones. This plays a vital role in preimplantation development studies.

The library preparation and quality control checks take 2 days. It is possible to pause the library preparation at several points of the protocol as well as to prepare multiple libraries in parallel. Guidelines for batch effect removal for accurate comparisons have been delineated if samples are processed in several libraries.^6^

### Institutional permissions

The mouse embryos were created under the ethical approval ID 1528 (Jose Inzunza) evaluated and accepted by Linköping’s Animal Experiment Ethics Committee.

### Workspace setup

The library preparation should be done in two separate areas for pre-PCR (until step 26) and post-PCR with separate consumables and small equipment in each area. This is necessary to avoid the contamination of the RNA as well as the presence of RNAase and DNAase in the pre-PCR area to ensure the good quality of mRNA. Cell lysis buffer and capture buffer should be prepared in pre-PCR area.

## Key resources table

**Table:** 

REAGENT or RESOURCE	SOURCE	IDENTIFIER
**Chemicals, peptides, and recombinant proteins**		

RiboLock Rnase inhibitor, 40 U/μL	Thermo Fisher	Cat# EO0382
SuperScript IV Reverse Transcriptase, 200 U/μL	Invitrogen	Cat# 18090050
dNTP mix 10 mM each	Thermo Scientific	Cat# R0192
NEBNext End Repair Enzyme	NEB	Cat# E6050S
*E. coli* DNA ligase	NEB	Cat# M0205S
NEBNext dA-Tailing Reaction Buffer, 10×	NEB	Cat# B6059S
Klenow exo, 5 U/μL	NEB	Cat# M0212S
Phusion Hot Start Flex 2× Master Mix	NEB	Cat# M0536S
T4 DNA ligase, 5 U/μL	Thermo Scientific	Cat# EL0014
Terra PCR Direct Polymerase Mix	Takara	Cat# 639270
DTT No-Weigh Format	Thermo Fisher	Cat#A39255
2 M KCL, RNAase free	Ambion	Cat#AM9640G
Tris 1 M, pH 8	Ambion	Cat# AM9855G
*KCl powder*	Sigma	Cat# P9541-500G
*Peg 8000*	Sigma	Cat# 81268
Peg 6000	Sigma	Cat# 528877-1KG
Triton X-100 10%	Sigma	Cat#93443-100ML
Tween-20	Fisher Scientific	Cat#BP337-100
Acid Tyrode’s solution	Sigma	Cat# T1788-100ML
Phosphate buffered saline (PBS)	Sigma	Cat# P5119-100ML
Nuclease-free water	Ambion	Cat#AM9937

**Critical commercial assays**		

ERCC RNA Spike-In Mix	Ambion	Cat#4456740
KAPA Library Quantification Kit	Roche	Cat#KK4824
NucleoSpin Gel and PCR Clean-up	Macherey-Nagel	Cat#740609.50
Dynabeads MyOne Carboxylic Acid beads	Invitrogen	Cat#65011
Dynabeads MyOne C1 Streptavidin	Invitrogen	Cat#65001
Agencourt AMPure XP	Beckman Coulter	Cat#A63881
NextSeq 500/550 High Output v2 kit	Illumina	Cat#FC-404-2005

**Oligonucleotides**		

Primers for library preparation	Table 1, This paper	N/A
STRT-T30 V-LNA	Table 1, This paper	N/A
Barcode primers (table 2)	IDT/Metabion	^1^

**Biological samples**		

Mouse embryos	Karolinska Institutet	C57BL/6

**Other**		

Thermocycler with a heated lid	N/A	N/A
Plate centrifuge	N/A	N/A
Vortex machine	N/A	N/A
Focused ultrasonicator for DNA fragmentation	BioRuptor Pico or compatible	N/A
Magnetic rack for 1.5 mL tubes	N/A	N/A
0.65 mL Microtubes for Bioruptor Pico	Diagenode	Cat# C30010011
DynaMag-96 Side Magnet	Invitrogen	Cat#12331D
Stereomicroscope	N/A	N/A
EZ-Grip pipettor	CooperSurgical	Cat# 7-72-2800
Eppendorf LoBind tubes	Eppendorf	Cat# 0030108035
Nunc® IVF multidish	Merck	Cat# Z688754-120EA
Thermal Mixer (Thermal Shaker) for 1.5 mL tubes	N/A	N/A
Bench Top Centrifuge for 1.5/2 mL tubes	N/A	N/A
VersiPlates, 96-well Low Profile PCR Strip Tube Plate	Thermo Scientific	Cat#AB-1800
VersiCap Mat, Domed Cap Stripes	Thermo Scientific	Cat#AB-1810
DNA LoBind Tubes 1.5 mL	Eppendorf	Cat#30108051
8-channel Multipipette	N/A	N/A
TapeStation (for quality control)	Agilent	N/A
D1000 ScreenTape	Agilent	Cat#5067- 5582
D1000 Reagents	Agilent	Cat#5067- 5583
D5000 ScreenTape	Agilent	Cat#5067-5592
D5000 Reagents	Agilent	Cat#5067-5593

**Software and algorithms**		

STRTN pipeline d0ba6a1	This paper	
STRT2 pipeline b3e589c	Ezer et al.^1^	https://github.com/my0916/STRT2
Conda v22.9.0	Anaconda Inc.^7^	https://www.anaconda.com/
Picard v2.18.29	Broad Institute^8^	http://broadinstitute.github.io/picard/
HISAT2 v2.2.1	Kim et al.^9^	https://daehwankimlab.github.io/hisat2/
SAMtools v1.6	Danecek et al.^10^	http://www.htslib.org/
BEDtools v2.30.0	Quinlan and Hall^11^	http://bedtools.readthedocs.io/
R-base v4.2.0	R Core Team^12^	https://www.r-project.org/
R Package: ggplot2 v 3.4.0	Wickham et al.^13^	https://ggplot2.tidyverse.org/
featureCounts v2.0.1	Liao et al.^14^	http://subread.sourceforge.net/
Seqtk v1.3	–	https://github.com/lh3/seqtk
UCSC Bedgraphtobigwig v377	Kent et al.^15^	https://github.com/bioconda/bioconda-recipes/tree/master/recipes/ucsc-bedgraphtobigwig
R package: ggbeeswarm v0.7.1	–	https://cran.r-project.org/web/packages/ggbeeswarm
R package: Seurat v3.0.2	Butler et al.^16^	https://satijalab.org/seurat/
StringTie v2.1.7	Shumate et al.^17^	https://ccb.jhu.edu/software/stringtie/
FastQC v0.11.9	Andrews^18^	https://www.bioinformatics.babraham.ac.uk/projects/fastqc/
MultiQC v1.13	Ewels et al.^19^	https://multiqc.info/

## Materials and equipment

**CRITICAL:** Prepare all the buffers and solutions in the pre-PCR area, preferably in a PCR workstation or RNA clean room, and use nuclease-free water. We advise that pre- and post-PCR areas are clearly separated, with the consumables and small equipment being also separated.

### Cell capture plate

**Timing: 30 min**

• To prepare cell lysis buffer mix the following in 1.5 mL LoBind tube:

**Table:** 

Reagent	Final concentration	Amount for 1 well
Nuclease free water		2.25 μL
dNTP mix	0.95 μM	1 μL
RiboLock	0.4 U/μL	0.1 μL
DTT	1.9 mM	0.04 μL
Tris pH 8.0	3.8 mM	0.04 μL
Triton X-100	0.04%	0.04 μL
STRT-T30 V-LNA	0.53 μM	0.53 μL
**Total**		**4 μL**

Store the plates with the cell lysis buffer at −20°C for 6 months.

• Prepare 96-well Low Profile PCR Strip Tube Plate (Versiplate) with 4 μL of cell lysis buffer in each well.
• We suggest using multichannel pipette

### Capture buffer for step 14

• Add 12.5 g of PEG 6000 in 50 mL tube and add 37 mL of 3 M KCl solution and 500 μL of 1 M Tris pH 8.
• Dissolve it under warm running water.

***Note:*** Store at 21°C–22°C for 6 months.

### ERCC spike-in control dilution (for step 7)

**Timing: 30 min**

• Make 1:10 dilutions and then 1:100 dilutions as working stocks of ERCC RNA Spike-In mix to avoid unnecessary thaw and freeze cycles.
• Make aliquots of 1.3 μL of 1:100 dilutions in 0.2 mL tubes and store at −80°C for up to 12 months.
• Before library preparation, take one 0.2 mL tube of 1:100 stock and add 130 μL of Buffer EB and mix well (to create 10,000× dilution).
• Take 1 μL of 10 000× dilution for each library master mix.

### Adapter cassette (20 μM) for step 53 based on Ezer et al. (2021)

**Timing: 1 h**

• Preheat the heat block on 72°C.
• In a 1.5 mL tube mix the following reagents:

**Table:** 

Reagent	Final concentration	Amount
Adapter-1 ssDNA (100 μM)	20 μM	20 μL
Adapter-2 ssDNA (100 μM)	20 μM	20 μL
KCl (2 M)	50 mM	2.5 μL
Nuclease-Free Water	N/A	57.5 μL
**Total**	**N/A**	**100** μL

• Place the tube on the heat block for 5 min.
• Turn off the heat block and let it cool slowly to 20°C–22°C.
• Make aliquots of 20 μL and store at -20°C (stable up to 12 months).
• Thaw on ice before each use.

### Barcoded primers for step 20 based on Ezer et al. (2021)

**Timing: 2 h**

• If barcoded primers are ordered in dry condition, dissolve each primer in nuclease free water to make 100 μM stocks (store at −20°C. Stable up to 12 months).
• Prepare 48 well plate with 10 μM barcoded primer solutions in numerical order by adding 10 μL of the primer in each well and 90 μL of nuclease free water. Store at −20°C (stable up to 12 months).
• Before library preparation, place the barcoded primers plate at +4°C to be thawed for 3–4 h.

### PCR2 primer mix for step 57 based on Ezer et al. (2021)

**Timing: 20 min**

• To prepare primer mix for the step 57, mix the following reagents in 1.5 mL tube:

**Table:** 

Reagent	Final concentration	Amount
primer PCR-2cF (100 μM)	10 μM	10 μL
primer PCR-2cR (100 μM)	10 μM	10 μL
Nuclease-Free water	N/A	80 μL
**Total**	**N/A**	**100 μL**

• Make aliquots of 20 μL and store at −20°C for 12 months.

### Buffer based on Ezer et al. (2021)

#### Buffer EB

• Mix the following reagents in the 50 mL falcon tube.

**Table:** 

Reagent	Final concentration	Amount
TrispH 8.0	5 mM	0.25 mL
Tween-20	0.02%	0.1 mL
Nuclease-Free water	N/A	49.65 mL
**Total**	**N/A**	**50 mL**

Store at 20°C–22°C up to 12 months.

• This buffer is used to make ERCC Spike-In dilution 1:10 000, for the step 34, and for step 42 and steps that follow.

## Step-by-step method details

Figure 1.

**Figure 1:**
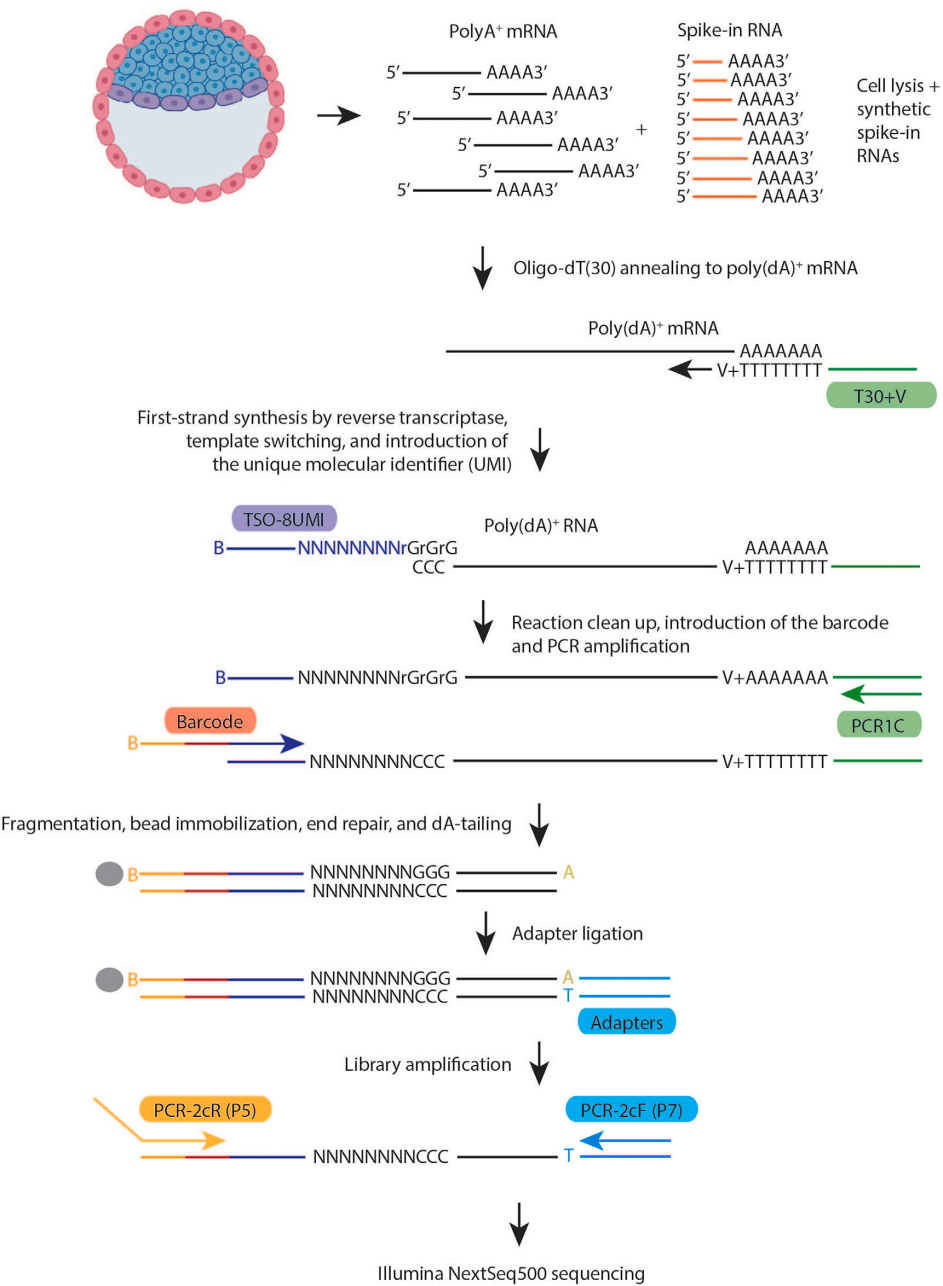
Overview of the step-by-step STRT-N library preparation Embryo is placed in the cell lysis buffer and mRNA is released. This mRNA is then mixed with reverse transcription reagents containing ERCC Spike-In RNA. First-strand cDNA synthesis is performed by capturing mRNA polyA tail with oligo dT(30)+V, and the mRNA is transcribed with the addition of 3 cytosine molecules on the 5′ end of mRNA. Oligo TSO-8UMI is used to bind to those cytosine molecules, promoting template switching and introduction of UMIs into the cDNA. cDNA is then cleaned, and the barcodes are introduced at the 5′ end. After the cDNA amplification, each sample is barcoded, and all the reactions can be pooled for the next steps which include purification, fragmentation, and adapter cassette ligation. The library preparation is finished by the further 5′ end amplification before sending the ready library for sequencing. Adapted from Ezer et al.^1^

### Embryo lysis

**Timing: 20 min**

This section describes the handling of the embryos and placing them in the cell lysis buffer.

**Figure 2:**
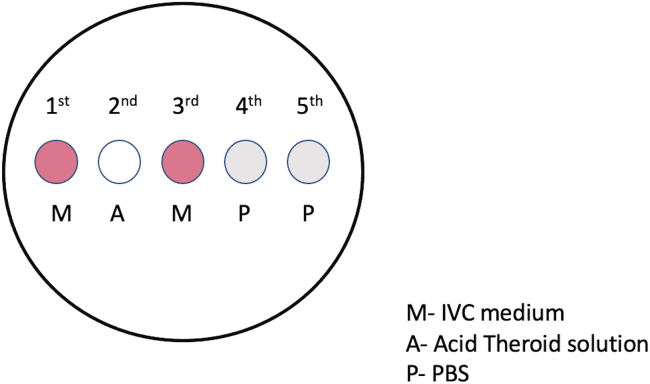
Graphical demonstration of the plate preparation for the removal of zona pellucida There are 5 drops, each 30 μL placed on a 60 mm embryo safe dish. First and third drop in pink color represent IVC medium, 2^nd^ drop is Acid Tyrode’s solution and 4^th^ and 5^th^ drops are PBS.
  c. Place embryo to the 1^st^ drop, then to 2^nd^ and watch the zona pellucida dissolves.
    i. transfer embryo to the 3^rd^ drop to wash out the acid.
    ii. transfer to the 4^th^ drop containing PBS.
    iii. Wash embryo again in the last PBS drop.
  d. Transfer the whole embryo with the least amount of PBS possible to one well of the low-profile PCR strip Versiplate.
  e. Place the strip on ice and spin it down in the plate centrifuge (30 s at 1500 × *g*) or on the table centrifuge.
  f. Place the strip/plate on dry ice immediately.
  g. Place the plate in the −80°C.

1. Embryo handpicking:
  a. Under stereomicroscope, take one embryo from the 4-well culture dish. using EZ-Grip pipettor for the embryos until blastocyst stage. For blastocyst stage we recommend 10/20 μL pipette.
  b. In 60 mm plate, place 5 drops of 30 μL each (Figure 2.): 1^st^ *in vitro* culture (IVC) medium; 2^nd^ is Acid Tyrode’s solution; 3^rd^ is IVC medium; 4^th^ and 5^th^ are PBS.

**CRITICAL:** It is essential that the plate is placed on dry ice as soon as possible after placing the embryos to the lysis buffer. The plate should be placed on dry ice even if the library will be prepared on the same day, as this step helps the cell lysis.

***Note:*** We used the whole embryos to create the library we demonstrated in this protocol. If you want to use embryonic single cells, embryo biopsy can be performed by zona drilling pipette and then blastomere extraction. Blastomere should then be placed into a PBS solution, hand-picked and transferred to one well in the cell lysis plate.

**Pause point:** Plates can be stored at −80°C for 12 months.

### Reverse transcriptase reaction (day 1)

**Timing: 2 h 30 min**

This is the first part of the protocol and describes the steps involved in capturing mRNA and performing the first DNA strand synthesis.

2. Take out the capture cell plate from the −80°C (previously stored for maximum of 6 months) and keep it on dry ice.
3. Preheat the PCR block to 80°C and transfer the plate directly from dry ice to the PCR block.
4. Hold hand on the plate lid, and once it is warm, close the lid of the PCR machine and denature RNA for 2 min (RNA Denaturation step).
5. Place the plate immediately on ice.
6. Prepare the reverse transcriptase mix using following reagents in 1.5 mL Eppendorf LoBind tube.

**Table:** 

RT mixture: Reagents	Stock	One rx	One library (56rx)
Nuclease-free water		2 μL	112 μL
PEG 8000 50% w/v	50%	1 μL	56 μL
5× SuperScript IV RT buffer	5×	2 μL	112 μL
DTT	100 mM	0.5 μL	28 μL
TSO	40 μM	0.53 μL	28 μL
RiboLock	40 U/ μL	0.25 μL	14 μL
SuperScript IV RT enzyme	200 U/ μL	0.25 μL	14 μL
**Total volume**		**6.5 μL**	**364 μL**

7. Add 1 μL of ERCC Spike-In mix (1: 10,000× dilution).
8. Vortex the mix and spin down quickly.
9. Divide the mixture to 8 tube strip for an easier pipetting of mixture to the plate.
10. Add 6.5 μL to each well of the plate using an 8-channel pipette.
11. Quickly vortex and spin down plate either in the plate centrifuge (20 s at 1500 × *g*) or spin the individual strips on the table centrifuge.
12. Place the plate in the PCR machine and start the reverse transcription (RT) reaction.

**Table:** 

STRT-N reverse transcription (lid 80°C, vol 10 μL)			
Steps	Temperature	Time	Cycles
Warm-up/hold	80°C	Hold	
RNA Denaturation (step 4)	80°C	2 min	
Loading	4°C	hold	
RT Reaction	42°C	60 min	
RT Reaction	50°C	2 min	10
RT Reaction	42°C	2 min	
Inactivation	85°C	5 min	
Hold	4°C	forever

13. Spin down the plate (1 min at 1500 × *g*).

**CRITICAL:** It is highly important that the RT reaction is mixed thoroughly before starting the PCR cycles.

### Clean up and full cDNA amplification (day 1)

**Timing: 4 h**

This section describes the clean up steps of reverse transcriptase reaction to remove all the unnecessary reaction elements and minimize the byproduct formations in the cDNA amplification, as well as introduction of barcoded primers to each sample and performing the cDNA amplification.

14. In a 1.5 mL tube mix 50 μL Dynabeads MyOne Carboxylic Acid beads and 400 μL capture buffer (previously stored for maximum of 6 months at 21°C–22°C)
15. Add 7 μL of magnetic beads and capture buffer mix to the RT product. Mix well by pipetting or quick vortex (vortex together with the plate holder, so that it reduces the liquid spilling to the caps).
16. Incubate for 15 min at room temperature (21°C–22°C) and place the plate on a 96 well magnetic rack for 3 min.
17. Remove all supernatant by pipetting.
18. Spin the plate or take strips apart and spin down on the table centrifuge and place back on the magnetic rack. Remove all traces of the supernatant.
19. Resuspend the beads in 22 μL nuclease-free water.
20. Add 1.5 μL of barcoded primers (10 μM) (previously stored at −12°C for up to 12 months).
21. Prepare PCR master mix in 2 mL Eppendorf DNA LoBind tube.

**Table:** 

PCR master mix: Reagents	One rx.	One library (56rx)
2× Terra Direct PCR buffer	25 μL	1400 μL
Terra Direct PCR polymerase mix (2 U/μL)	1 μL	56 μL
10 μM PCR-1c primer	0.5 μL	28 μL
**Total volume**	**26.5 μL**	**1484** μL

22. Vortex thoroughly and spin down the tube.
23. Add 26.5 μL to each well, mix well by pipetting or vortex quickly.
24. Start the PCR.

**Table:** 

STRT-N PCR cycling conditions			
Steps	Temperature	Time	Cycles
Initial Denaturation	98°C	2 min	1
Denaturation	98°C	10 s	2
Annealing	55°C	30 s	
Extension	68°C	5 min	
Denaturation	98°C	10 s	23
Annealing	60°C	30 s	
Extension	68°C	5 min	
Final extension	72°C	10 min	1
Hold	4°C	forever

25. Spin down the evaporation drops either in plate centrifuge (1 min at 3000 × *g* or on the table centrifuge).

**Figure 3:**
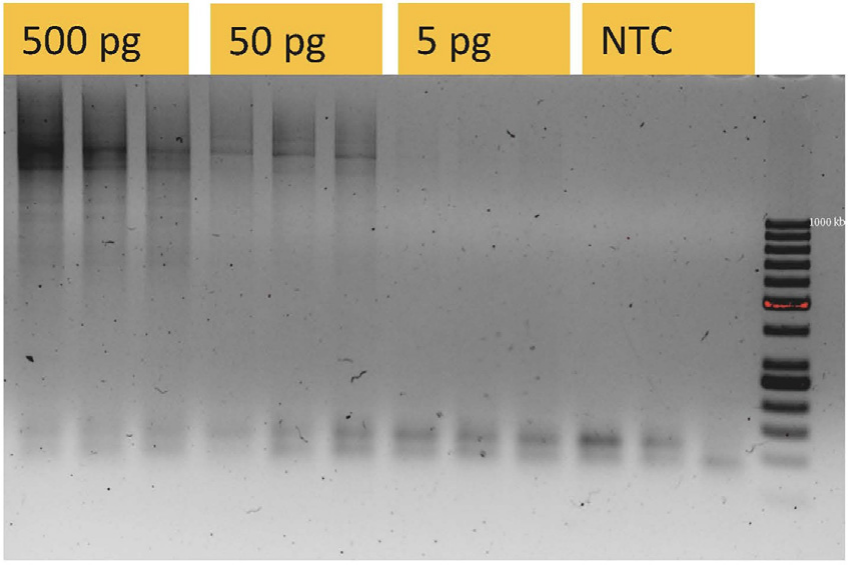
cDNA visualized on 2% TAE gel After cDNA amplification in step 25 it is possible to visualize the product on the 2% TAE gel. You should observe a smear representing a variety in the length of the cDNA. In this figure you can see the difference between the strength of the smear based on the amount of initial RNA input going from 500 pg in the first 3 lanes, 50 pg in the second 3 lanes to single cell level (5 pg) in the next 3 lanes. Non template control (NTC) should not have the smear. Ladder is 1 kb.

***Note:*** It is possible to visualize the cDNA products on a 2% TAE gel, if the library isn’t planned to be sequenced (Figure 3).

### Sample pooling and purification (day 1)

**Timing: 30 min**

This section describes pooling together all the samples to one reaction as they have already been barcoded in the previous steps and purifying the reaction.

26. Place the plate on the 96 well magnetic rack and wait for 3 min for beads to bind.
27. Collect the clear supernatants into six 1.5 mL LoBind Tubes. Purify the library with PCR Clean-up column (NucleoSpin Gel and PCR Clean-up/Macherey-Nagel- https://www.mn-net.com/media/pdf/02/1a/74/Instruction-NucleoSpin-Gel-and-PCR-Clean-up.pdf)

***Note:*** Purification should be done according to the manufacturer’s instructions, except for the step 4: silica membrane should be centrifuged for 2 min instead of 1 min; for step 5: preheat the elution buffer provided in the kit at 70°C for 5 min.

28. Elute the cDNA twice using 16 μL each time of the previously heated elution buffer. At the end there should be 32 μL of cDNA in the clean 1.5 mL LoBind tube.
29. Take out 2 μL of the library for the 1^st^ quality check with TapeStation- QC1. There is now 30 μL of library left for the next step.

**Pause point:** At this point in experiment, it is possible to pause by placing the library at -20°C for up to 1 month.

### Fragmentation (day 2)

**Timing: 40 min**

This section described the fragmentation of DNA to achieve the wanted length (300-350bp) of DNA that can be sequenced on the Illumina platform.

30. AMPure XP beads should be mixed well: the solution should be brown and homogeneous.
31. Take 24 μL of AMPure XP beads and add them to the 30 μL of cDNA from step 29. The beads to sample ratio is 5:4.
32. Mix well and incubate at room temperature (21°C–22°C) for 10 min, before placing the tube to the magnetic rack for 3 min.
33. Remove the supernatant and then remove the tube from the magnetic rack.
34. Re-suspend the beads in 100 μL Buffer EB (previously stored for maximum of 6 months at 21°C–22°C), mix well, and incubate at RT for 1 min.
35. Place the tube back on the magnetic rack to capture the beads. After 2 min: transfer the 100 μL supernatant containing cDNA to a 0.65 mL BioRuptor shearing microtube.
36. Quickly spin down the tube and place it on ice for 10 min.
37. Fragmentation program is 10 cycles 30 s ON, 30 s OFF; target 350 bp (we used BioRuptor sonicator machine from 2013-2019).
38. Purify the fragmented cDNA with the PCR Clean-up column (NucleoSpin Gel and PCR Clean-up/Macherey-Nagel- https://www.mn-net.com/media/pdf/02/1a/74/Instruction-NucleoSpin-Gel-and-PCR-Clean-up.pdf) according to the manufacturer’s protocol.

***Note:*** Purification should be done according to the manufacturer instructions, except for the step 4: silica membrane should be centrifuged for 2 min instead of 1 min.

39. Elute the cDNA with 32 μL of elution buffer, provided in the kit, and take 2 μL aside for the 2^nd^ quality check with TapeStation (QC2).

**CRITICAL:** It is important that while assembling the holder for sonication, 11 extra 0.65 mL BioRuptor shearing microtubes containing 100 μL of water are placed together with the one tube containing 100 μL of cDNA, to ensure the equivalent sonication.

***Note:*** Fragmentation step can be done using other compatible machines such as Covaris Focused ultrasonicator, by using 130 μL microtubes and target 300 bp using manufacturer’s protocol.

**Pause point:** At this point in experiment, it is possible to pause by placing the library at −20°C for up to 1 month.

### End-repair, adapter ligation and 5′end amplification (day 2)

**Timing: 4 h**

This section describes the preparation of DNA for the sequencing. Following the fragmentation, the DNA is cut at the random points, ends of the DNA must be repaired to be able to ligate the adaptors needed for sequencing. In this section the Buffer EB is used, please make sure it’s freshly made every 6 months and stored at room temperature (21°C–22°C).

40. Add 12 μL of MyOne C1 Streptavidin beads to the 30 μL of fragmented cDNA from step 39, mix well by pipetting and wait for 10 min.
41. Place the tube on the magnetic rack until solution is clear. Remove and discard the supernatant.
42. Take the tube off the magnetic rack and wash the beads by adding 200 μL of Buffer EB. Mix the beads well by pipetting and place the tube back to the magnetic rack. Wait until solution is clear and remove the supernatant.
43. Repeat the washing (step 42) one more time.
44. Re-suspend the beads in 16 μL of Buffer EB.
45. Add the following reagents:

**Table:** 

Reagent	Amount
Beads containing cDNA in Buffer EB (from step 44)	16 μL
NEBNext End Repair Buffer, 10×	2 μL
NEBNext End Repair Enzyme Mix	1 μL
*E.coli* ligase	1 μL
**Total**	**20 μL**

46. Place the tube on the thermal shaker, incubate for 20 min on 500 rpm at RT (21°C–22°C).
47. Place the tube on the magnetic rack until solution is clear, remove the supernatant.
48. Wash the beads 2 times, by repeating step 42 twice.
49. Re-suspend the beads in 17 μL of Buffer EB and add the following reagents:

**Table:** 

Reagent	Amount
Beads containing cDNA in Buffer EB (from step 49)	17 μL
NEBNext dA tailing buffer, 10×	2 μL
Klenow exo- (5 U/μL)	1 μL
**Total**	**20 μL**

50. Place the tube on the thermal shaker, incubate for 20 min on 500 rpm at 37°C.
51. Place the tube on the magnetic rack until solution is clear, remove the supernatant.
52. Wash the beads 2 times, by repeating the step 42 twice.
53. Re-suspend in 18 μL of Buffer EB. Add the following reagents:

**Table:** 

Reagent	Amount
Beads containing cDNA in Buffer EB (from step 53)	18 μL
T4 DNA Ligase buffer (10×)	3 μL
Adapter cassette (20 μM)	1 μL
PEG-4000 (50%)	6 μL
T4 DNA ligase (5 U/μL)	2 μL
**Total**	**30 μL**

54. Do not vortex, only mix gently by pipetting, incubate on the bench for 60 min at RT and mix gently every 20 min.
55. Place the tube on the magnetic rack, until the solution is clear and remove supernatant.
56. Perform beads washing with 200 μL of Buffer EB twice.
57. Re-suspend the beads in 20 μL of Buffer EB and add the following:

**Table:** 

Reagent	Amount
Beads containing cDNA in Buffer EB (from step 57)	20 μL
Phusion Hot master mix, 2×	25 μL
PCR-2 primer mix (10 μM each)	5 μL
**Total**	**50 μL**

58. Transfer 50 μL to 0.2 mL PCR tube and start the KIN-P2 program.

**Table:** 

KIN-P2 PCR cycling conditions			
Steps	Temperature	Time	Cycles
Initial Denaturation	98°C	1 min	1
Denaturation	98°C	10 s	12 cycles
Annealing	62°C	20 s	
Extension	72°C	20 s	
Final Extension	72°C	1 min	1
Hold	4°C	infinite	

### Final purification (day 2)

**Timing: 20 min**

This section describes the final library purification needed to remove the unwanted residues of the primers and reaction elements before the library can be sequenced.

59. Take out the 0.2 mL tube from the PCR machine and transfer everything to a 1.5 mL LoBind tube. Place the tube on a magnetic rack. Wait until the supernatant, that now contains the PCR products, is clear and transfer it, to a clean 1.5 mL LoBind tube.
60. Add 45 μL of AMPureXP beads and mix extensively by vortexing.
61. Incubate for 10 min on the bench (21°C–22°C), place on the magnetic rack and wait until the solution is clear. Remove and discard the supernatant.
62. Spin down the tube for 10 s on a mini-centrifuge to collect down the drops and place back on the magnetic rack
63. Remove all the traces of supernatant.
64. Re-suspend the beads in 20 μL of Buffer EB, mix well by pipetting, and wait for 3 min.
65. Place back on the magnetic rack until the solution is clear. Collect the solution (ready library) and transfer it to a new 1.5 mL LoBind tube.
66. Take out 2 μL for the final quality check (QC-3).

**Pause point:** Store the sequencing ready library at −20°C for up to 6 months.

### Library quality control (day 3)

Before the library can be send for the sequencing the final quality control should be performed to determine the library concentration which is essential for the sequencing.

67. Check the concentration of the ready library from step 66 on the TapeStation D1000 ScreenTape system. The concentration of the ready library should be 2–30 nM and peak should be around 300 bp (see Figure 4 for expected quality check results).

**Figure 4:**
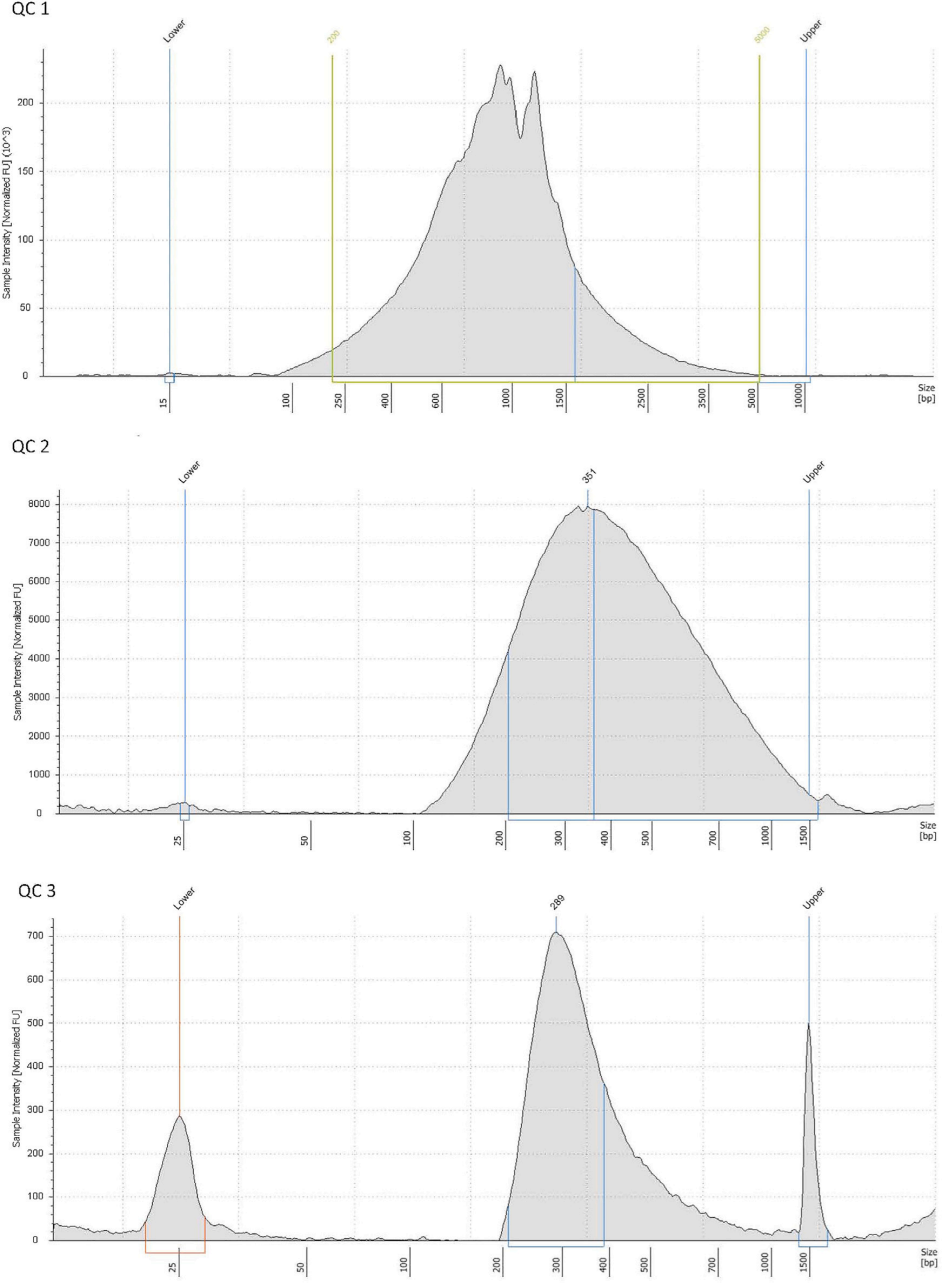
Quality controls of the library using TapeStation In step 29, QC1 is performed using HS D5000 ScreenTape. You should see similarly to the gel visualization, a variety of transcripts in the size range of 200 bp to >2000 bp long. In step 39, QC3 is performed after fragmentation. As protocol is aiming for the fragments that are 350bp long, you should observe a wide peak around 350 bp. In step 66, QC3 is performed, and you should see a sharp peak around 350 bp.
68. The concentration of the library should be checked with the KAPA Library Quantification Kit according to the manufacturer instructions. (https://pim-eservices.roche.com/eLD/api/downloads/ca670ceb-fb38-eb11-0291-005056a71a5d?countryIsoCode=pi).

**CRITICAL:** It is important that the correct library concentration is determined before sequencing as the volume of library loaded for the sequencing is decided based on that. If the concentration between the TapeStation and KAPA kit measurement is different, we have relied on the results provided by KAPA kit.

### Sequencing (day 3)

Sequencing of STRT-N libraries can be performed on the Illumina NextSeq System. While read 1 is 75+ cycles of non-index read, read 2 must be exactly 6 cycles of index read as the sample barcode is 6 bp. Download the base call (BCL) files; demultiplexing and conversion to FASTQ files are unnecessary. The location of the “BaseCalls” folder in the files will be specified as BaseCallsDir_PATH at step 72. “RunInfo.xml” in the BCL files records the number of cycles.

***Note:*** We usually sequence the libraries using NextSeq 500 with custom primer STRT2seq (Table 1) and High Output v2.5 kit, 75 cycles at the Biomedicum Functional Genomics Unit (FuGU). In the BCL files, there should be the “BaseCalls” folder at Data/Intensities. Although we use the 75 cycles kit, 86 and 6 cycles of sequencing are performed for reads 1 and 2 respectively. Therefore, we usually describe the logical structure as 8M3S75T6B (for step 72a; 8 + 3+75 = 86); change the structure when the length of read 1 is shorter than 86 cycles. The read1 sequencing read length is 86 bp while the read2 sequencing length is 6 bp.

**Table 1:** STRT-N primers

Primer name	Sequence (5′-3′)	Note
TSO-8 UMI	Biotin-ACGACGCTCTTCCGATCTNNNNNNNNrGrGrG	
T30-V-LNA	TTAAGCAGTGGTATCAACGCAGAG TCGACTTTTTTTTTTTTTTTTTTTTTTTTTTTTT+V	PAGE PURIFICATION
PCR1c primer	AAGCAGTGGTATCAACGCAGAGT	
adapter1	CAAGCAGAAGACGGCATACGAGATT	
adapter2	Phos-ATCTCGTATGCCGTCTTCTGCTTG	
PCR-2cF	CAAGCAGAAGACGGCATACGAGATT	
PCR-2cR	AATGATACGGCGACCACCGAGATC TACACCACGTCTGAACTCCAGTCAC	
Index Custom primer	CGA GAT CTA CAC CAC GTC TGA ACT CCA GTC AC	

### Preparations for data processing

This section includes the minimal hardware requirements, installing conda packages and pipelines as well as details of required genome indexes to be processed by STRT-N bioinformatics data processing pipeline. The installation of conda packages and pipeline and running the STRT-N pipeline should be run on Linux terminal. We tested the pipeline on Windows Subsystem for Linux running Ubuntu 20.04.3 in a Windows 11 laptop, and on the Puhti Linux cluster at the IT Center for Science (CSC) services (https://docs.csc.fi/computing/systems-puhti/).

### Hardware requirements

Building genome indexes (step 71), sequence data processing (step 72), visualization on UCSC (step 73–74) and visualization using Seurat (step 75) require about 150GB, 15GB, 50MB and 150MB of memory depending on genome size and raw data size.

### Installing packages with conda and pipelines

This section includes the installation of required dependencies and pipeline installation.

69. Create conda environment, install the required software packages in the yaml file and activate them as following.

conda env create -n STRTN-test -f STRTN-env.yml

conda activate STRTN-test

***Note:*** In this example, the required software packages will be prepared the environment named “STRTN-test”, but you may change it to any name according to your project.

70. Install the main pipeline and visualization of data pipeline from the following GitHub repository.

git clonehttps://github.com/gyazgeldi/STRTN.gitSTRTN-test

cd STRTN-test

***Note:*** In this example, the pipeline will be cloned into the “STRTN-test” folder, but you may change it to any name according to your project.

### Preparing genome indexes and sequence dictionary

**Timing: ∼3 h using 8 CPU cores**

71. Prepare HISAT2 genome indexes and sequence dictionary in a platform which has big memory to rapidly and efficiently access reference sequence information. Here, we illustrate the case for house mouse genome mm39.
  a. Obtain the genome sequences of reference and ERCC spike-ins.
    wgethttps://hgdownload.soe.ucsc.edu/goldenPath/mm39/bigZips/mm39.fa.gz
    unpigz -c mm39.fa.gz | ruby -ne '$ok = $_ !∼ /ˆ>chrUn_/ if $_ =∼ /ˆ>/; puts $_ if $ok' > mouse_reference.fasta
    wgethttps://tsapps.nist.gov/srmext/certificates/documents/SRM2374_putative_T7_products_NoPolyA_v2.FASTA
    cat SRM2374_putative_T7_products_NoPolyA_v2.FASTA >> mouse_reference.fasta
  b. Extract splice sites and exons from the annotation file using HISAT2 (v2.2.1)^9^
    Here we used wgEncodeGencodeBasicVM30 as the annotation file.
    wgethttps://hgdownload.soe.ucsc.edu/goldenPath/mm39/database/wgEncodeGencodeBasicVM30.txt.gz
    unpigz -c wgEncodeGencodeBasicVM30.txt.gz | hisat2_extract_splice_sites.py - | grep -v ˆchrUn > splice_sites.txt
    unpigz -c wgEncodeGencodeBasicVM30.txt.gz | hisat2_extract_exons.py - | grep -v ˆchrUn > exons.txt
  c. Build the HISAT2 indexes of the mouse reference genome and ERCC Spike-in RNAs with hisat2-build function.
    hisat2-build mouse_reference.fasta --ss splice_sites.txt --exon exons.txt mouse_index/mouse_reference
  d. Create the sequence dictionary for the reference and Spike-in sequences with Picard (v2.18.29) (https://github.com/broadinstitute/picard) CreateSequenceDictionary function.
    picard CreateSequenceDictionary R=mouse_reference.fasta O=mouse_reference.dict
  e. Put the genome indexes, genome fasta file, sequence dictionary to same folder.

mv mouse_reference.dict mouse_reference

mv mouse_reference.fasta mouse_reference

***Note:*** The genome indexing step requires large memory and it might not be possible to carry out it on a laptop. Therefore, we created genome indexes for the house mouse genome mm39 using wgEncodeGencodeBasicVM30 annotation file in Puhti. The details of procedure is in https://github.com/gyazgeldi/STRTN#how-to-build-hisat2-index-in-csc and code is available in https://doi.org/10.5281/zenodo.7524239. The built indexes can be accessed in https://doi.org/10.5281/zenodo.7457660.

### Sequence data processing

**Timing: ∼15 h using 8 CPU cores**

**Figure 5:**
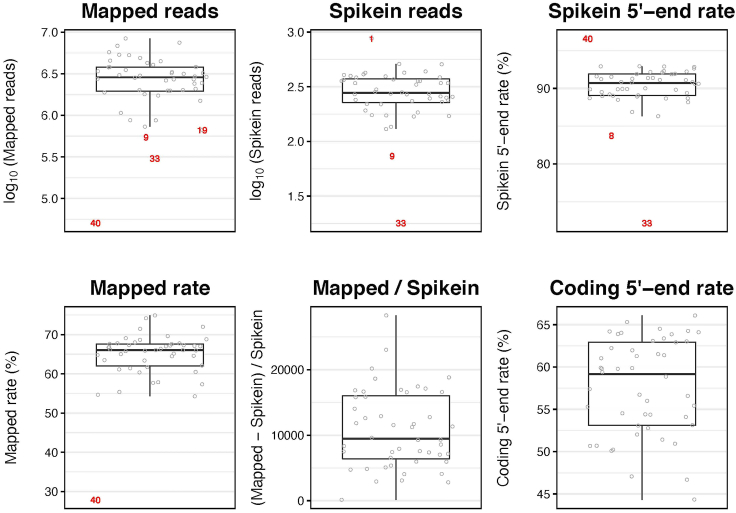
Output from the sequence analysis Mapped reads, Spike-In reads, Spike-In 5′ end rate, mapped rate, mapped/Spike-In, coding 5′ end rate from a successful library containing 47 RNA samples of mouse embryos at different developmental stages and one non-template control (NTC). We removed outliers that are samples 1, 8, 9, 19, 33, and 40 for further steps.
iii. Read count table output, named as OUTPUT_byGene-counts.txt
iv. Filtering summary, OUTPUT_byGene-counts.txt.summary
v. Resulting BAM files (.bam)
vi. Index files (.bai) of the resulting BAM files
vii. Metrics file indicating the number of matches/mismatches between the barcode reads and the actual barcodes is shown per lane, named as ExtractIlluminaBarcodes_Metrics
viii. Alignment summary, named as HISAT2_Metrics
ix. Metrics file indicating the numbers of duplicates, named as MarkDuplicates_Metrics
x. BAM files containing reads except for duplicate and non-primary reads, named as OUTPUT.output.bam
xi. BigWig files for each strands of each sample, named as OUTPUT_plus.bw and OUTPUT_minus.bw
xii. BigBed file for coding-5′end annotation file, named as coding coding_5end.bb

***Note:*** Spike-in_5end_rate represents the proportion of STRT reads aligned to the 5′ end (50 nt) of spike-in RNAs. And coding-5end_rate represents the proportion of STRT reads aligned within the 5′ UTR of the protein-coding genes and the proximal (500 nt) upstream. These values must be high and stable enough, as a decrease in the values shows poor library synthesis quality. However, low or unstable coding-5end_rate is sometimes acceptable when it is expected, for example in the study of preimplantation embryos in which maternal transcripts are degrading. The assessment of whether values are good or bad depends on factors such as sample type and quality. For example, we found the spike-in 5′ end rate is 85%–95%, while the coding 5′ end rate is 45%–65% in a successful mouse library.

This section includes the commands to run the main pipeline on a desktop computer. For CSC users, the main pipeline can be accessed in https://github.com/gyazgeldi/STRTN/blob/master/STRTN-CSC.sh. Overall, the main pipeline automatically processes raw sequenced data file with these steps: demultiplexing, read mapping, marking duplicates, annotation, quality check, quantification and creating files for visualization. The pipeline then automatically gives following output files:

i. Quality check report for all samples, named as OUTPUT-QC.txt
ii. Quality check report by boxplots indicating Mapped_reads, Mapped_rate, Spikein_reads, Mapped / Spikein, Spikein-5end_rate, and Coding-5end_rate for all samples to check quality of reads and to mark outlier samples with red characters (Figure 5), named as OUTPUT-QC-plots.pdf

72. Run the STRT-N main pipeline with the following command (see an example run). Please make sure that barcode sequence with barcode name (Table 2^1^) is prepared as barcode.txt.
  Usage:
  ./STRTN.sh -o {OUTPUT_NAME} -g {GENOME_VALUE} \
  -a {ANNO_VALUE} -b {BaseCallsDir_PATH} -i {Index_PATH} -w {WorkingDir_PATH} \
  -c {center_VALUE} -r {run_VALUE} -s {READ_STRUCTURE}
  Example run:
  ./STRTN.sh -o STRTN_MOUSE_LIB -g mm39 -a wgEncodeGencodeBasicVM30 \
  -b /mnt/c/Users/gamyaz/STRTN-Pipeline/Data/Intensities/BaseCalls \
  -i /mnt/c/Users/gamyaz/STRTN-Pipeline/mouse_index/mouse_reference \
  -w /mnt/c/Users/gamyaz/STRTN-Pipeline \
  -p /mnt/c/Users/gamyaz/STRTN/ENTER/pkgs/picard-2.27.4-hdfd78af_0/share/picard-2.27.4-0 \
  -c FUGU -r RUNBARCODE -s 8M3S75T6B
  Required and optional arguments:
  {output} defines the prefix of output files such as STRTN_MOUSE_LIB.
  {reference_genome} is the reference genome such as mm39.
  {annotation} is the gene annotation used for quality check and counting. For mm39, ref (RefSeq) or wgEncodeGencodeBasicVM∗ or kg (KnownGenes) can be chosen. See also the GitHub repository’s README.md file for details.
  {path/to/BaseCalls} is the path to the Illumina basecalls directory.
  {path/to/index} is the directory and basename of the HISAT2 index for the reference genome.
  {path/to/working} is the working directory.
  {Sequencing_center} is the name of the sequencing center that produced the reads.
  {Run_barcode} is the barcode of the run. Prefixed to read names.
  {picardhome} is the path to picard.jar at conda packages folder.
  Here, we describe Step-by-step methods from demultiplexing, read mapping, marking duplicates, annotation, quality check, quantification to creation files for visualization as well as optional analyzes.
  a. To correctly separate and analyze the sequencing data based on barcodes, the barcodes are prepared, the number of lanes are determined and the necessary parameters file for barcodes are created. To understand which sequences came from which samples, the sequencer raw base call data files are demultiplexed with Picard ExtractIlluminaBarcodes and IlluminaBasecallsToSam. This step processes raw base call and related files which are binary reporting (InterOp) and cluster location (LOC) and creates unaligned BAM files based on the well-specific barcodes (Table 2^1^).
    ***Note:*** It is necessary to convert the sequencer output format to the downstream formats like SAM/BAM by defining the read structure. The input data consists of 92 base clusters (cycles) and we described this logical structure as 8M3S75T6B: 8 cycles (bases) of molecular barcode, 3 cycles skipped, 75 cycles of the template, and 6 cycles of sample barcode.
  b. To align reads to GRCm39 reference genome and ERCC Spike-in RNAs (SRM 2374) [NIST SRM 2374 Certificate of Analysis, https://www-s.nist.gov/srmors/certificates/2374.pdf, 2013] with the Gencode annotation file (wgEncodeGencodeBasicVM30) as a guide of exon junctions. The unaligned BAM files are sorted and converted to FASTQ files with Picard SamToFastq and aligned using HISAT2.
  c. To generate a unique molecular identifier (UMI)-annotated BAM files, the aligned BAM files are merged with the original unaligned BAM files by Picard MergeBamAlignment.
  d. To merge all lanes, UMI-annotated BAM files corresponding to each sample derived from four lanes are merged using Picard MergeSamFiles and to mark the potential PCR duplicates, Picard MarkDuplicates is used.
  e. To check the quality of reads, information on genomic regions is extracted from annotation files. After that, the resulting BAM files per sample are indexed and the reads that do have not primary alignment and PCR duplicate flags 256 and 1024 are removed using SAMtools (v1.6).^10^ Reads mapped to protein-coding genes and spike-in RNAs and 5′ ends of them, are counted using BEDtools (v2.30.0).^11^ The values of i) log10 of total mapped read counts, ii) mapping rates, iii) log10 of Spike-in RNA read counts, iv) ratios of total mapped read counts versus Spike-in RNA read counts, v) 5′ end capture rates of Spike-in RNAs, and vi) 5′ end capture rates of protein-coding genes are plotted with R (4.2.0)^12^ package ggplot2 (v3.4.0)^13^ the outlier samples are marked with barcode numbers (Figure 5). Please consider these outlier samples for the further downstream analysis in accordance with your scientific question. As an example, we decided to remove outlier samples from the successful mouse library to make a reliable data representation because these samples might have degraded.
  f. To quantify gene expression level, Subread featureCounts (2.0.1)^14^ is used to count reads align to 5′end of genes with parameters '-s 1 -largestOverlap -ignoreDup -primary'. In this step, uniquely mapped reads within 5′ UTR or 500 bp upstream of the protein-coding genes and the first 50 bp of spike-in sequences are counted.
  g. To visualize read alignments, resulting output BAM files are used. The BAM files are indexed and the reads that do have not primary alignment and PCR duplicate flags 256 and 1024 are removed using SAMtools with below commands for each sample.
  h. To visualize the number of reads as a continuous signal, BigWig files are created. First, BedGraph files are created from BAM files by calculation of scale factor with running bin/calculationScaleFactor.R for each sample using bedtools genomecov. Followed by, the BedGraph files are converted into BigWig files for forward and reverse strands for each sample with size files using bedGrapthToBigWig.
  i. To visualize annotation items, BigBed file from coding-5′-end.bed file is created. The output coding-5′-end BED file is sorted and converted into BigBed file with the chromosomal size file using bedToBigBed with below commands.
    ***Optional:*** Moreover, one can perform as abovementioned gene-based analysis by running STRTN.sh to find only reads within the 5′ UTR or the proximal upstream of protein-coding genes. Besides, one can perform TFE (transcript far 5′ end)-based analysis by running STRTN-TFE.sh. This analysis is developed originally by Töhönen et al.,^20^ and is modified to run in CSC and on a laptop based on TFE analysis in Ezer et al.^1^ Here, TFEs are defined as the first exon (5′ end region) of assembled STRT-N reads mapped to reference genome using StringTie (v2.1.7)^17^ and assigned with unique IDs and gene expression levels quantified as described above. The details are available at https://github.com/gyazgeldi/STRTN/blob/master/STRTN-TFE-README.md.
    ***Note:*** If TFE-based analysis is planned to perform, please make sure to add the option '-dtá (downstream-transcriptome-assembly) for the main pipeline STRTN.sh or STRTN-CSC.sh, which is required in the HISAT2 alignment process, it helps the transcript assembly by StringTie.
    ***Optional:*** One can perform sequence quality check analysis of sequenced data by running fastq-fastQC.sh or fastq-fastQC-CSC.sh. Here, the output BAM files are converted into fastq files (without duplicated reads) that can be submitted to public sequence databases. Followed by, FASTQC files are generated for each fastq file, and based on the FASTQC results, MultiQC report is generated.

### Visualization the results in UCSC genome browser

**Timing: ∼30 min**

This section describes how to visualize the results on genome browsers, for example UCSC Genome Browser tool^21^ or Integrative Genomic Viewer (https://igv.org/). Hereinafter, we describe steps for visualization of data in UCSC using BAM files for alignments, BigWig files for read frequency and BigBed file of coding-5′ end for annotation items. The detailed procedure of visualization pipeline is in https://github.com/gyazgeldi/STRTN/blob/master/Visualization-in-UCSC-README.md. Overall, the pipeline takes resulting BAM files, BigWig files and BigBed file of coding-5′ end as the inputs and gives fully automatically following output files.

i. Hub file containing required parameters in UCSC, named as hub.txt
ii. The link indicating hub.txt location that can be copied directly to Track Hub in the UCSC genome browser
73. To visualize with required parameters such as type, visibility etc. hub.txt file is created for each data format types. For forward and reverse strands, color parameters are set up. As an example, first samples for each data formats are viewable. See an example hub.txt file in https://doi.org/10.5281/zenodo.7603268.
74. To display these created BAM, BigWig and BigBed files (see step 72g-i) in UCSC Genome Browser, these files must be uploaded with hub.txt file to a hosting service (https://genome.ucsc.edu/goldenPath/help/hgTrackHubHelp#Hosting).

***Note:*** We uploaded the files to Allas container using swift protocol and the pipeline gives automatically sharable hub.txt link that can be directly copied to Track Hub in UCSC. The code is available in STRTN-UCSC-Allas.sh (https://github.com/gyazgeldi/STRTN/blob/master/STRTN-UCSC-Allas.sh). As an example, we visualized the data using this link: https://a3s.fi/swift/v1/AUTH_2698cec7f280451ebad2e7a291561bec/STRTN_MOUSE_LIB-HUB/hub.txt.

### Visualization the results using Seurat

**Timing: ∼1 min**

**Figure 6:**
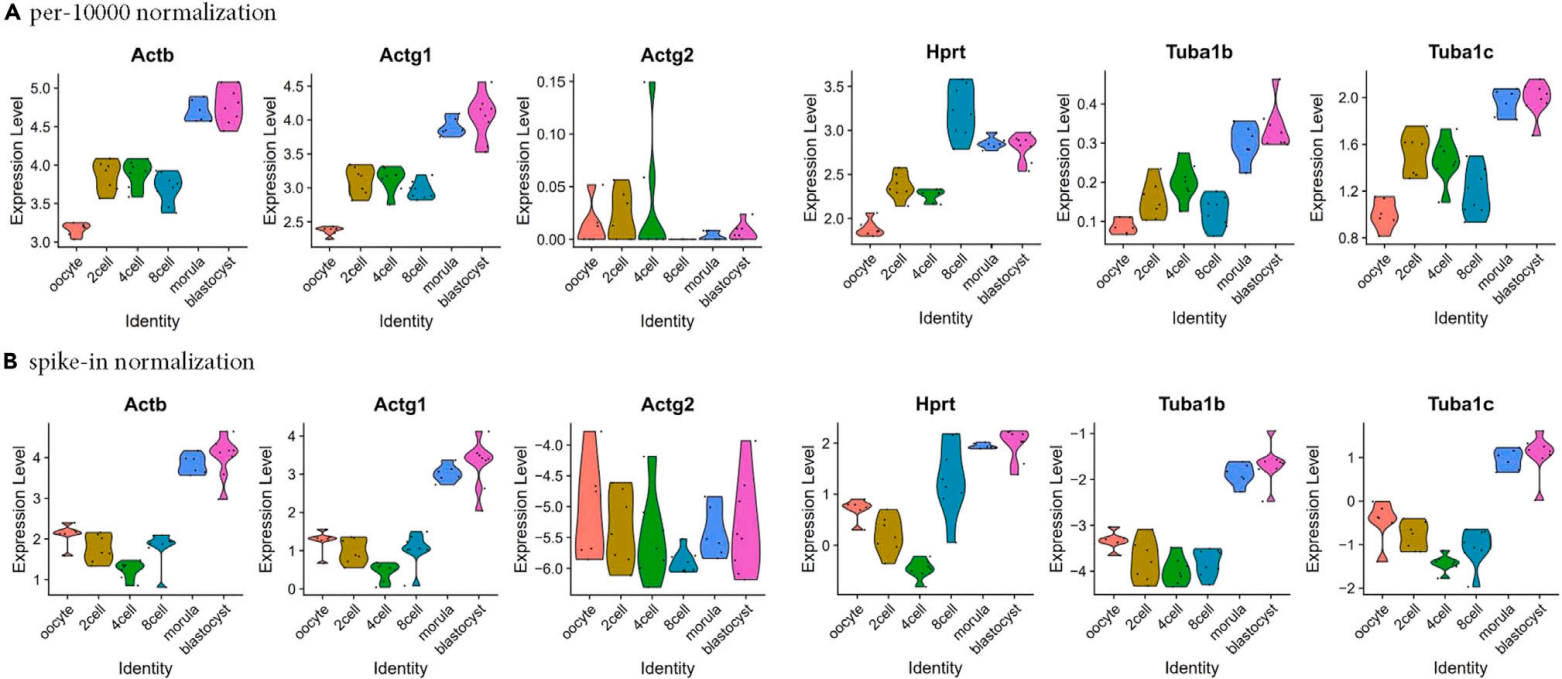
Differences in the expression profiling for 6 genes between the two ways of normalizing the samples: per-10000 normalization or Spike-In based normalization (A) violin plots obtained based on per-10000 normalization of 6 genes in early embryo development; (B) violin plot obtained based on Spike-In normalization.

**Figure 7:**
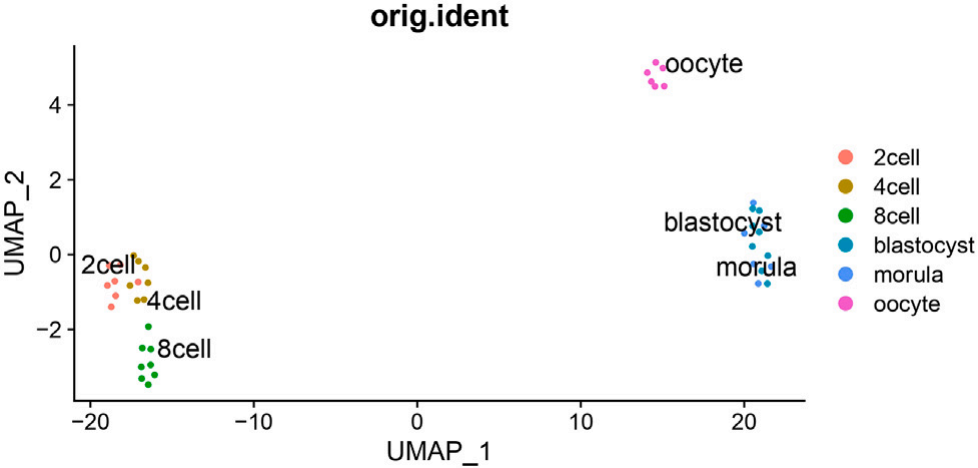
Output from single-cell RNA sequence analysis UMAP plot representation of a successful library, showing the developmental stages from 42 RNA samples.

This section includes details for analysis of STRT-N library results using Seurat package (v3.0.2)^16^ by running STRTN-Seurat.sh from normalization, data reduction to plotting using R language or R platform. The details of procedure are available in https://github.com/gyazgeldi/STRTN/blob/master/STRTN-Seurat-README.md. Overall, the pipeline takes quality check values file, count matrix file and sample explanation file and gives fully automatically following output files.

i. Beeswarm plots indicating the visualization quality check values for each developmental stage
ii. Output R plots containing Elbow, JackStraw, PCA, UMAP and violin plots as the examples from successful STRT-N mouse library
75. The resulting output count matrix is used to create Seurat object with CreateSeuratObject function and the data is normalized based on spike-in normalization with NormalizeData function. We normalized measured values based on spike-in normalization because in preimplantation development, RNA amount is changing at the different stages according to ref.^22^ Hereby, the default per-million based normalization is invalid for this STRTN library analysis. Therefore, we added +1 value to all expression values and divided the values by sum of spike-ins expression values (see below). Differences between the expression profiling with spike-in normalization and without spike-in normalization is demonstrated in Figure 6. We highly recommend spike-in normalization to enables accurate comparisons of expression levels between samples. Followed by, as an example, we visualized resulting data from mouse STRT-N library (Figure 7). The example usage is available in: https://github.com/gyazgeldi/STRTN/blob/master/STRTN-Seurat.sh. See example of R-output files in https://doi.org/10.5281/zenodo.7603268.

strt.seurat.obj <- NormalizeData(strt.seurat.obj, normalization.method = "LogNormalize", scale.factor = 10000)

reads.p1 <- cts + 1

reads.p1.spikes <- colSums(reads.p1[1:97,])

strt.seurat.obj[["RNA"]]@data <- as(as.matrix(log(reads.p1/rep(reads.p1.spikes, each=nrow(reads.p1)))), "dgCMatrix")

## Expected outcomes

Expected results of the library quality control check points can be observed in the Figure 4. The concentration of the ready library should be 2–30 nM.

Figure 3 shows how cDNA products look on 2% TAE gel after the cDNA amplification step 24. There should be smear on the gel.

From one sequencing kit, we obtained 181,843 - 12,756,053 reads per sample with a median of 4,205,390. After excluding duplicated reads, we obtained 50,793–8,392,691 reads per sample with a median of 2,436,566 for a mouse library (2.0 pM of library and 20% of PhiX). The values of log10 of total mapped read counts, log10 of Spike-in RNA read counts, 5′ end capture rates of Spike-in RNAs, mapping rates, ratios of total mapped read counts versus Spike-in RNA read counts and 5′-end capture rates of protein-coding genes are expected at least 6.0, 2.5, 80%, 40%, 5000 and 50% as respectively depending on annotation status of relevant model organism. These values can be found in a plot in the out directory named as OUTPUT-QC-plots.pdf. The expected plot can be observed in Figure 5, obtained from a successful library. In addition, we obtained 14,469 protein-coding gene that have at least five mapped reads based on gene-based analysis from this library. While 15,031 protein-coding gene and non-protein coding gene that have at least five mapped reads were obtained after excluding intronic and unannotated TFEs based on TFE-based analysis from this library.

## Limitations

This method is ideal for the samples with very limited availability, such as mammalian embryos. The library consists of only 48 samples; thus, it is not suitable for cell lines from which enough RNA can be extracted. In this case bulk STRTseq would be better 3 if the aim of your study is looking at the different TSS, the STRTseq is superior in the coverage of 5′ end as shown in Abugessaisa et al.^23^

In addition, as this is 5′ end method, data about splice isoforms and 3′ UTR isoforms are not available.

## Troubleshooting

### Problem 1

Low cDNA concentration in QC1.

### Potential solution

Make sure that all the MyOne Carboxylic acid beads are well resuspended in the water before adding the barcoded primers and PCR mix. In case the beads are stuck to the walls of the well, we recommend spinning down the plate before resuspending the beads.

### Problem 2

Dominant peak at around 100 bp and little or no other size products in QC1.

### Potential solution

During the PCR the primers can form dimers that can take over the reaction.

• Make sure that supernatant in step 17 is removed completely.
• If this peak is not dominant and there is also enough cDNA, then the results will not be affected as these smaller products will be removed in the purification steps.

### Problem 3

No wide peak around 350 bp in QC2.

### Potential solution

It is possible that fragmentation didn’t work efficiently. It is necessary to repeat the fragmentation step as follows:

• Measure the exact amount of cDNA that is left after taking the 2 μL for QC2.
• Add AMPure XP beads to the sample in the ratio of 0.65× (this is to capture the larger fragments that haven’t been fragmented), mix well and incubate for 10 min on the bench.
• Put the tube on the magnetic rack and wait for 3 min until solution is clear.
• Transfer the supernatant to different tube (this is to protect already fragmented shorter products from over fragmenting them) and place the tube in the fridge until the fragmentation of longer products has been done again.
• Remove the tube from the magnetic rack and add 100 μL of Buffer EB and wait for 1 min.
• Place the tube again on the magnetic rack and wait until solution is clear.
• Transfer this solution to new 0.65 mL BioRuptor microtube and perform the fragmentation following the steps 36 and 37.
• Before purifying the content as in step 38, mix the solution from the tube that you kept in the fridge that contains shorter products with the solution in the 0.65 mL BioRuptor tube.
• Perform the clean up as in step 38 and repeat QC2.

### Problem 4

The concentration of the final library is low.

### Potential solution

The final library amplification (step 58) the number of PCR cycles can be increased by 1–2 more cycles.

### Problem 5

The mean quality value of base positions at the end of the read is low.

### Potential solution

If the mean quality value of base positions at the end of the read is low in the sequence quality histogram in the MultiQC report, reads can be trimmed by re-defining the read structure to use in demultiplexing step using Picard tool.

### Problem 6

Low Spike-In concentration in the sequencing library.

### Potential solution

Spike-In concentration should not be lower than 1000 copies per sample. We recommend the range to be between 1000-10 000 per sample. Based on this, calculate the initial Spike-In dilution that you will add to the library.

## Resource availability

### Lead contact

Further information and requests for resources and reagents should be directed to and will be fulfilled by the Lead Contact Nina Boskovic (nina.boskovic@ki.se).

### Materials availability

This study did not generate any new reagents.

## Data Availability

The raw data (BCL files) have been deposited to Zenodo: https://doi.org/10.5281/zenodo.7549819. The accession number for the FASTQ reported in this paper is available in EMBL’s European Bioinformatics Institute (EMBL-EBI)-BioStudies: S-BSST976. The code generated during this study is available in Zenodo: https://doi.org/10.5281/zenodo.7524239 and GitHub: https://github.com/gyazgeldi/STRTN. The built indexes can be accessed at GitHub: https://doi.org/10.5281/zenodo.7457660.
